# Endobronchial ultrasound versus conventional transbronchial needle aspiration in the diagnosis of mediastinal lymphadenopathy: a meta-analysis

**DOI:** 10.1186/s40064-016-3348-1

**Published:** 2016-10-05

**Authors:** Jun-Hong Yan, Lei Pan, Xiao-Li Chen, Jian-Wei Chen, Li-Ming Yan, Bao Liu, Yong-Zhong Guo

**Affiliations:** 1 Department of Ultrasonography, Binzhou Medical University Hospital, Binzhou, 256603 China; 2 Department of Respiratory and Critical Care Medicine, Binzhou Medical University Hospital, Binzhou, 256603 China; 3 Department of Critical Care Medicine, Jining First People’s Hospital, Jining, 272001 China; 4 Department of Infection Management, Binzhou Medical University Hospital, Binzhou, 256603 China; 5 Department of Respiratory and Critical Care Medicine, Henan Provincial People’s Hospital, Zhengzhou University, Zhengzhou, 450003 China; 6 Department of Respiratory Medicine, Xuzhou Central Hospital, The Affiliated Xuzhou Center Hospital of Nanjing University of Chinese Medicine, 199 South Jiefang Road, Xuzhou, 221009 Jiangsu China

**Keywords:** Transbronchial needle aspiration, Endobronchial ultrasound, Diagnostic yield, Mediastinal lymphadenopathy, Meta-analysis

## Abstract

Currently, whether endobronchial ultrasound-guided transbronchial needle aspiration (EBUS-TBNA) is superior to conventional TBNA (cTBNA) in the diagnosis of mediastinal lymphadenopathy remains controversial. We undertook a meta-analysis of randomized controlled trials (RCTs) to evaluate the diagnostic yield of EBUS-TBNA versus cTBNA in the diagnosis of mediastinal lymphadenopathy, both in benign and malignant etiologies. Computer-based retrieval was performed on PubMed and EMBASE. The quality was evaluated according to the quality assessment of diagnostic accuracy studies-2, and Meta-Disc was adopted to perform meta-analysis. The pooled sensitivity, specificity, and diagnostic odds ratio (DOR) with 95 % confidence intervals (CIs) were calculated. The summary receiving operating characteristic curve as well as the areas under curve (AUC) was measured. Four studies with a total of 440 patients met the inclusion criteria. Our results showed that the pooled sensitivity was 0.90 (95 % CI 0.85–0.94) and 0.76 (95 % CI 0.68–0.82), pooled specificity was 0.75 (95 % CI 0.60–0.87) and 0.94 (95 % CI 0.86–0.98), DOR was 75.38 (95 % CI 16.38–346.97) and 108.17 (95 % CI 13.84–845.35), and AUC was 0.9339 and 0.9732 for EBUS-TBNA group and cTBNA group, respectively. Although EBUS-TBNA with a higher sensitivity performs better than cTBNA, there is lack of enough evidence regarding EBUS-TBNA being superior to cTBNA in the diagnosis of mediastinal lymphadenopathy. Considering the limitations of methodology and limited data, further robust RCTs are needed to verify the current findings and investigate the optimal choice in patients receiving TBNA.

## Background

 Mediastinal lymphadenopathy include a variety of benign and malignant conditions, such as lung cancer and sarcoidosis, which are depended on the pathology in terms of the gold standard for diagnosis. Hence bronchoscopy still plays an important role in the diagnosis of mediastinal lymphadenopathy (Zaric et al. 2013). Conventional bronchoscopy including mucosal biopsies, brushings, lavage cytology, bronchoscopy transmural lung biopsy (TBLB), and transbronchial needle aspiration (TBNA), can achieve most of the clinical diagnosis for clinicians. In the 1980s, Wang et al. (1983) using a flexible bronchoscopy firstly improved the TBNA technology including the operating needle and ways, which thus promoted a widespread application of TBNA. Conventional TBNA (cTBNA) is performed to aspirate tissue from thoracic masses and pathological lymph nodes through a specially designed needle (Wang 1994), which is a well-established diagnostic method and widely used in cytological evaluation of thoracic mass lesions. With the development of the ultrasound technique, real time endobronchial ultrasound-guided (EBUS)-TBNA was introduced into clinical practice by Yasufuku and colleagues in 2004 (Yasufuku et al. 2004a, b), which has been widely used in the staging and diagnosis of mediastinal and hilar lymph node (Herth et al. 2006; Varela-Lema et al. 2009; Medford et al. 2010).

EBUS-TBNA seems to be the optimal choice because of real-time visualization, imaging of surrounding vessels and size of the target lesion during the sampling procedure (Medford 2011). Several studies showed that EBUS-TBNA has high sensitivity, specificity and safety in the diagnosis of sarcoidosis and staging of lung cancer (Adams et al. 2009; Gu et al. 2009; Varela-Lema et al. 2009; Yang et al. 2014; Trisolini et al. 2015). In a recent overview comparison of different staging modalities for non-small cell lung carcinoma, suggested that the pooled sensitivity for cTBNA and EBUS-TBNA was 78 and 89 %, respectively (Vander Laan et al. 2014). However, other studies suggested that EBUS-TBNA was not superior to cTBNA in lung cancer patients with mediastinal nodes real-time sampling (Bellinger et al. 2012; Jiang et al. 2014). To our knowledge, the clinical choice between EBUS-TBNA and cTBNA in patients with mediastinal lymphadenopathy still remains controversial. Furthermore, the diagnostic yield of EBUS-TBNA versus cTBNA in mediastinal lymphadenopathy has not yet been well established. Therefore, we pre-stated rigorous inclusion criteria and enrolled available randomized controlled trials (RCTs) combining EBUS-TBNA and cTBNA to critically assess the diagnostic yield of EBUS-TBNA versus cTBNA in the diagnosis of mediastinal lymphadenopathy.

## Methods

### Search strategy and selection criteria

Computer-based retrieval was performed on PubMed and EMBASE (up to Mar 2016) for eligible trials with the following keywords: “endobronchial ultrasound” and “transbronchial needle aspiration”. Eligible trials limited with randomized controlled trial and written in English were included. Only published trials were included. Bibliographies of all potential studies, including reference lists, citation searches, and relevant systematic reviews, were manually searched.

The following selection criteria were included: (1) population: consecutive patients (age > 18 years) with mediastinal lymphadenopathy undergoing TBNA; (2) study design: RCT comparing the diagnostic value of real time EBUS-TBNA versus cTBNA in the detection of mediastinal lymphadenopathy; (3) sufficient data: reported data allowing calculation of the true-positive (TP), false-positive (FP), false-negative (FN) and true negative (TN) values; and (4) reference standard: histopathological and/or cytological analysis, or close radiological and clinical follow up for at least 6 months after TBNA, as the reference standard.

### Data extraction and quality assessment

All data were extracted from all trials by two independent investigators (LP and BL). The data included first author, publication year, country, number of patients, age and sex of patients, needle type, sedation setting, primary results, and adverse events. Disagreements among authors were settled by discussion or a third investigator (XLC).

The quality of RCTs was evaluated according to the quality assessment of diagnostic accuracy studies-2 (QUADAS-2) (Whiting et al. 2011). The QUADAS-2 tool contains four key domains: (1) patient selection, (2) index test, (3) reference standard, and (4) flow and timing. Each domain is assessed as “yes”, “unclear”, and “no” to judge risk of bias. Furthermore, the first 3 domains are also assessed as “high”, “Unclear”, and “low” concern to judge applicability. We rated the quality assessment and risk of bias using the Revman 5.2.0 (Nordic Cochrane Centre).

### Statistical analysis

The present study was conducted and reported in accordance with the Preferred Reporting Items for Systematic Reviews and Meta-Analyses statement (Liberati et al. 2009). The DerSimonian-Laird random-effects model (DerSimonian and Laird 1986; Reitsma et al. 2005) was used to calculate the data as forest plot of pooled sensitivity, specificity, positive likelihood ratio (PLR) and negative likelihood ratio (NLP), and diagnostic odds ratio (DOR) with 95 % confidence intervals (CIs) for EBUS-TBNA and cTBNA, respectively. The summary receiving operating characteristic (SROC) curve and the pooled diagnostic accuracy (Q* index) as well as the areas under curve (AUC) were also measured. The SROC curve moves closer to the upper left corner of the larger area under the curve, which seems that the accuracy of diagnostic tests is higher. Z-test was performed to determine whether the sensitivity, specificity, and Q* index of EBUS-TBNA was significantly different from those of cTBNA. Heterogeneity was evaluated using the *I* ^*2*^ statistics, and threshold effect was determined using the spearman correlation coefficient (Higgins and Thompson 2002; Higgins et al. 2003). If *I* ^2^ > 50 %, potential sources of heterogeneity were identified by sensitivity analyses, which were conducted by omitting one study in each turn and investigating the influence of a single study on the overall pooled estimate. Furthermore, subgroup analyses were performed to explore observed heterogeneity and examine the influence of various exclusion criteria based on sample sizes (>60 vs. ≤60), region (Asia vs. North America), and patients with diagnosis either sarcoidosis or other thoracic lesions. All meta-analyses were performed using Meta-DiSc 1.4 (XI Cochrane Colloquium; Barcelona, Spain) (Zamora et al. 2006). A two-sided *P* value of <0.05 was regarded as indicate statistical significance.

## Results

### Bibliographic search results

**Fig. 1:**
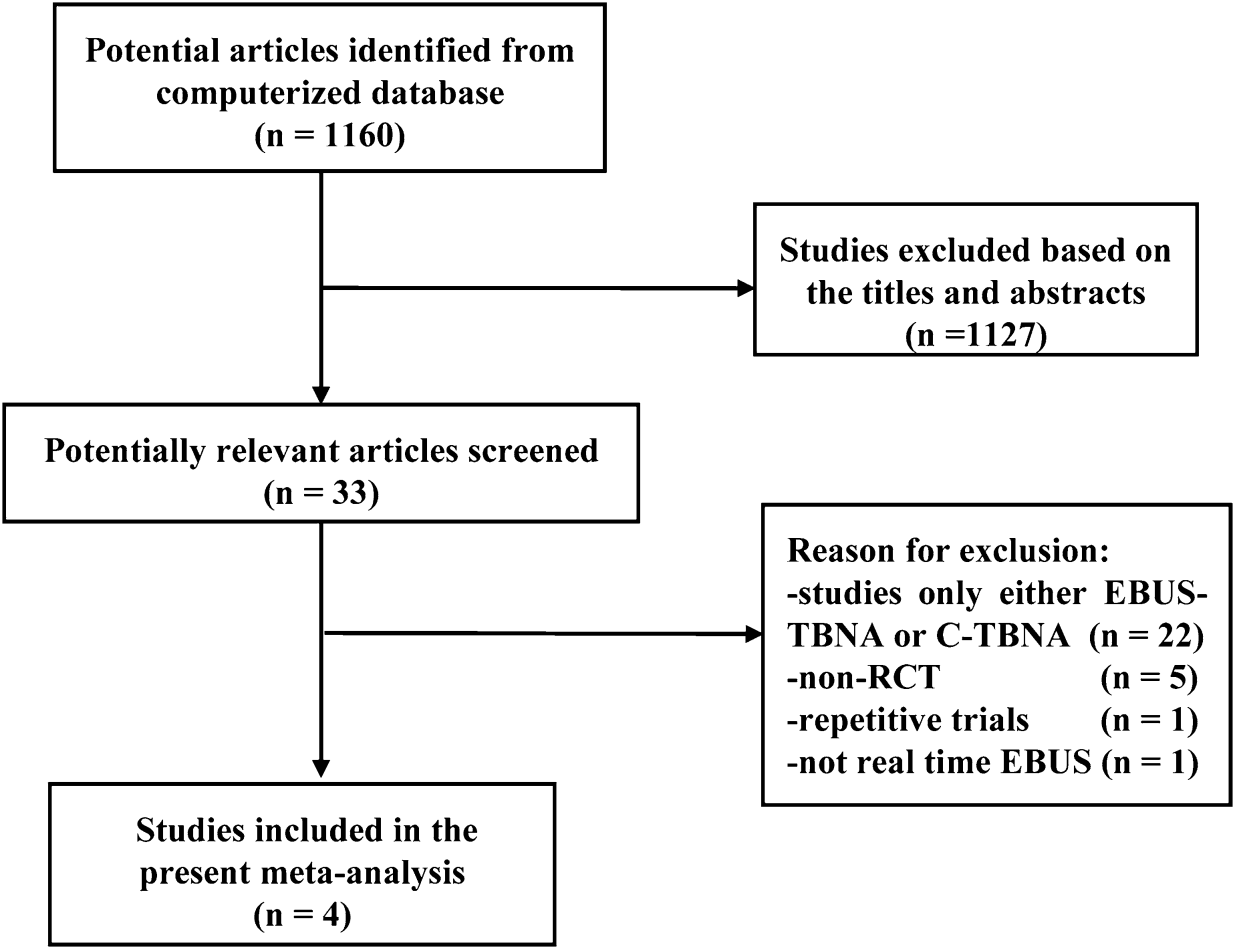
PRISMA flow diagram. *RCT* randomized controlled trial

A total of 1160 relevant articles were identified from the initial search. After reviewing the titles and abstracts, 1155 were excluded for duplicate studies and various reasons (e.g., case reports, editorials, reviews, non-randomized control trials, and or not using both EBUS-TBNA and cTBNA). A detailed flowchart for the study selection is presented in Fig. 1. Finally, the remaining 4 eligible RCTs with a total of 440 patients were identified for the present meta-analysis (Arslan et al. 2011; Gupta et al. 2014; Tremblay et al. 2009; Herth et al. 2004).

### Baseline characteristics and quality assessment

The main characteristics of the retrieved RCTs are shown in Table 1. From Table 1, we found that the sample size of 4 trials ranged from 50 to 200 and these RCTs were published between 2004 and 2014. Four RCTs involving 440 patients were conducted in 4 countries including Turkey (Arslan et al. 2011), India (Gupta et al. 2014), American (Herth et al. 2004), and Canada (Tremblay et al. 2009). Two RCTs enrolled patients with clinical diagnosis or suspected of sarcoidosis that had enlarged lymph nodes >1 cm (Tremblay et al. 2009; Gupta et al. 2014), and patients with other thoracic lesions that had at least one mediastinal node ≥0.7 (Herth et al. 2004) and 2 cm (Arslan et al. 2011) in short-axis diameter on computed tomography were included in the remaining two RCTs. Furthermore, all bronchoscopies were performed by experienced pulmonologists or by fellows under direct supervision. No rapid on-site evaluation (ROSE) was used in 4 RCTs. Additionally, the type of sedation was not the same settings in the four RCTs.

**Table 1:** Characteristics of randomized controlled trials included in the meta-analysis

Author country	Patients no. (E/C)	Age (years), Mean (SD), (E/C)	EBUS- versus Ctbna (Male/Female)	Study population (setting)	Operator	Needle type (E/C)	Sedation setting (E/C)	Primary results	Adverse events
Arslan et al. (2011) Turkey	60 (30/30)	57.33 (16.91)/54.97 (13.74)	26/4 versus 22/8	Patients with enlarged mediastinal lymph nodes that were defined as ≥2 cm (short axis) on CT	Both EBUS and cTBNA were performed by pulmonologists	21-gauge/21-gauge	All patients were under general anesthesia or conscious sedation	The diagnostic yield of EBUS-TBNA was higher than that of cTBNA in mediastinal lymph nodes (*P* = 0.872)	No complications were observed with the use of EBUS and/or cTBNA
Gupta et al. (2014) India	130 (62/68)	44.1 (10.8)/42.9 (11.5)	26/36 versus 27/41	Patients with clinical diagnosis of sarcoidosis that had enlarged right paratracheal and subcarinal lymph nodes >1 cm (short axis) on CT	All bronchoscopies were performed by experienced faculty or by fellows under direct supervision	21-gauge/21-gauge	Intravenous midazolam and pentazocine/None	EBUS-TBNA had the highest diagnostic yield than cTBNA in sarcoidosis (*P* = 0.004)	Three patients (1 with cTBNA and 2 with EBUS-TBNA) had minor bleeding, but no major adverse events happened
Herth et al. (2004) American	200 (100/100)	Total age: 51.9 (22.6)	61/39 versus 64/36	Patients with enlarged mediastinal lymph nodes that were defined as ≥0.7 cm (short axis) on CT	Both procedures were performed by pulmonologists	22-gauge/22-gauge	All patients were under general anesthesia or conscious sedation	EBUS-TBNA was superior to Ctbna in stations other than the subcarinal space in subcarinal lymph nodes (*P* < 0.001)	No complications were observed during and after EBUS and/or cTBNA
Tremblay et al. (2009) Canada	50 (24/26)	39.5 (8.6)/40.8 (12.8)	19/7 versus 14/10	Patients with suspected sarcoidosis that had pathologic mediastinal or hilar adenopathy (short axis, >1 cm) on CT	All bronchoscopies were performed by the interventional pulmonary medicine fellows	22-gauge/19-gauge	All patients were under conscious sedation	EBUS-TBNA was superior to cTBNA in mediastinal lymph nodes (*P* < 0.05)	Only two patients with moderate bleeding were seen following cTBNA

**Fig. 2:**
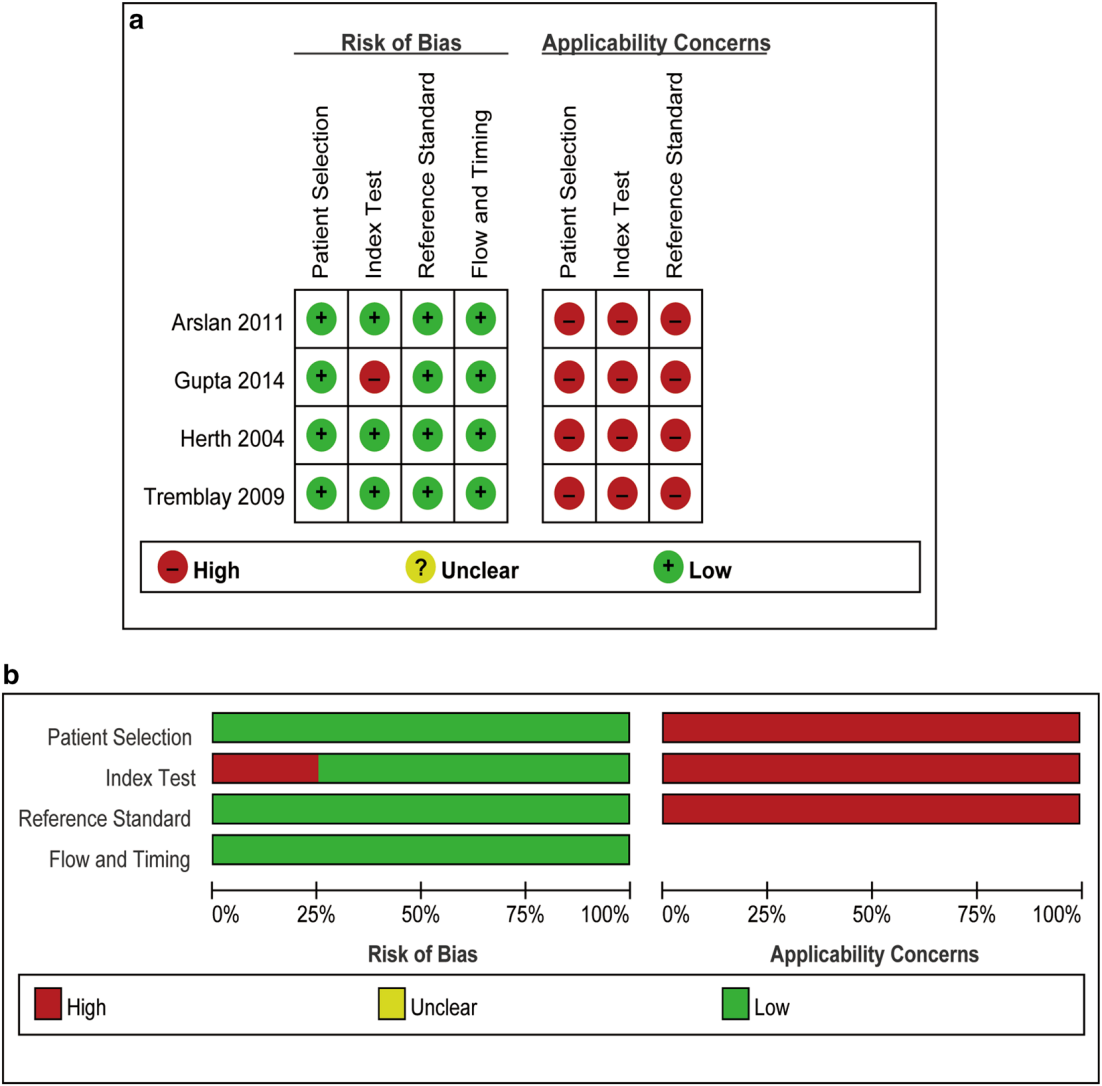
Study quality assessment by the quality assessment of diagnostic accuracy studies-2 criteria: **a** Risk of bias and applicability concerns summary: review authors’ judgements about each domain for each included study; **b** Risk of bias and applicability concerns graph: review authors’ judgements about each domain presented as percentages across included studies

Two authors (LP and BL) agreed on each item of the QUADAS-2. The risk-of-bias analyses suggested that all trials were followed at low risk in terms of patient selection, index test, reference standard, and flow and timing except only one RCT (Gupta et al. 2014) with a high risk of the index test. In addition, all trials were followed in high concern regarding applicability. The detailed quality assessment of 4 RCTs was illustrated in Fig. 2.

### Diagnostic value of EBUS-TBNA and cTBNA

**Fig. 3:**
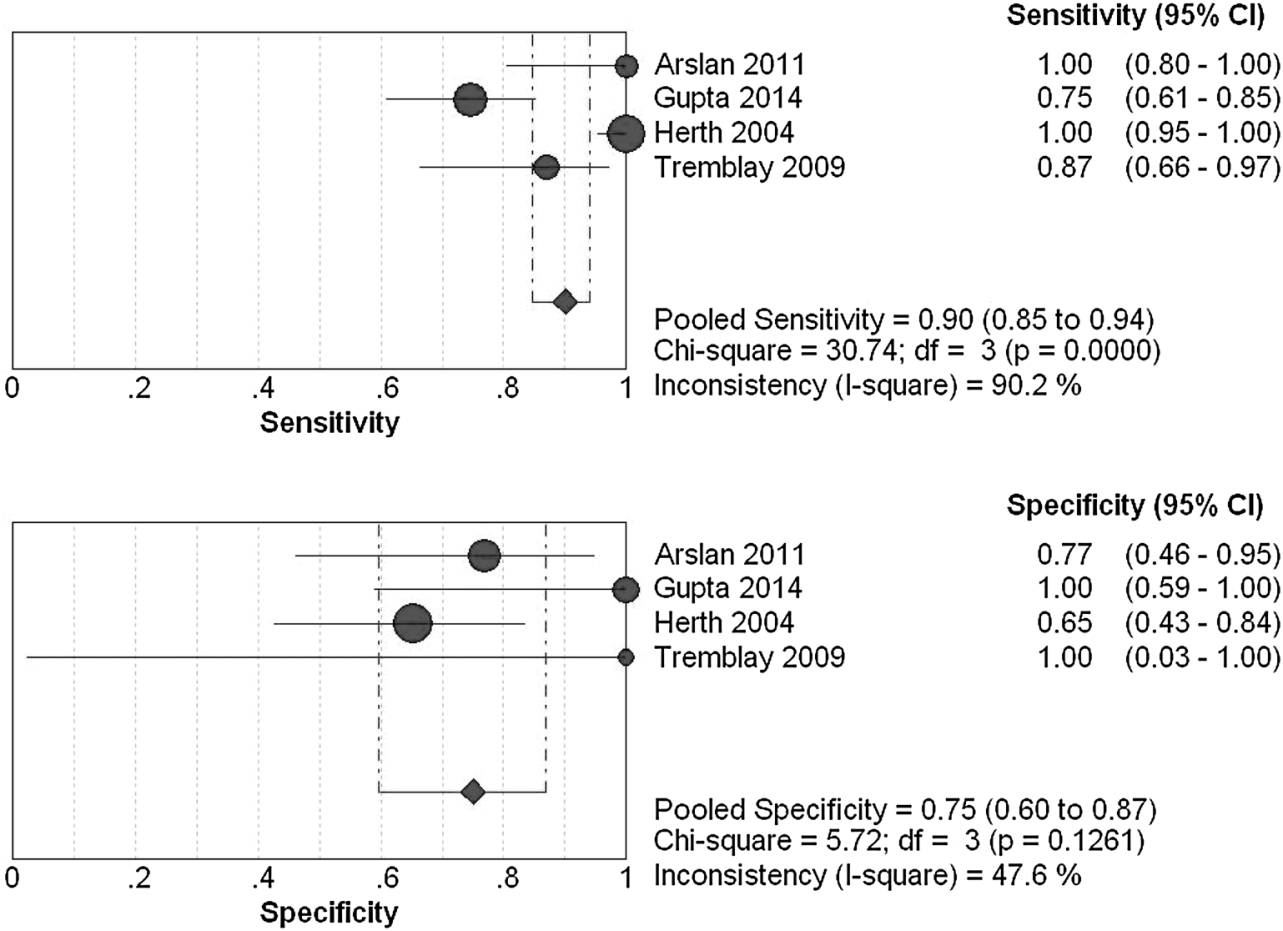
Forest plots of the pooled sensitivity and specificity for EBUS-TBNA by the random-effects model

**Fig. 4:**
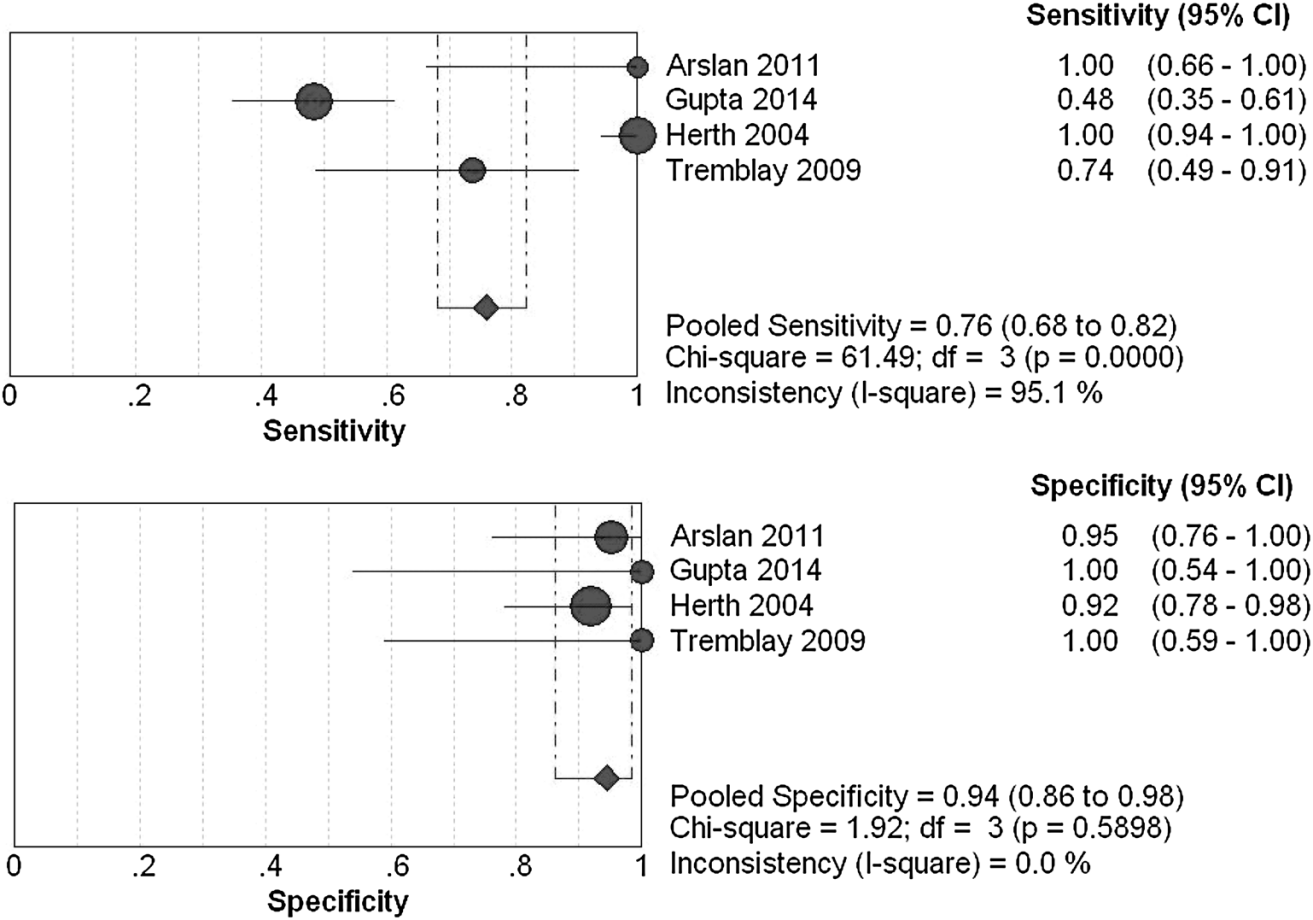
Forest plots of the pooled sensitivity and specificity for cTBNA by the random-effects model

The overall diagnostic sensitivity was 0.91 (95 % CI 0.85–0.94; *χ* ^*2*^ = 32.36; *I* ^*2*^ = 90.7 %) and 0.76 (95 % CI 0.68–0.82; *χ* ^*2*^ = 61.49; *I* ^*2*^ = 95.1 %), specificity was 0.92 (95 % CI 0.78–0.98; *χ* ^*2*^ = 6.61; *I* ^*2*^ = 54.6 %) and 0.94 (95 % CI 0.86–0.98; *χ* ^*2*^ = 1.92; *I* ^*2*^ = 0.0 %) for EBUS-TBNA group (Fig. 3) and cTBNA group (Fig. 4), respectively. Heterogeneity was significant in terms of the pooled sensitivity for two arms. Next, sensitivity analyses were performed to further explore potential source of heterogeneity across studies. Further exclusion of any single study did not resolve the heterogeneity, and the pooled sensitivity ranged from 0.82 (95 % CI 0.73–0.89; *χ* ^*2*^ = 9.05; *I* ^*2*^ = 77.9 %) to 0.97 (95 % CI 0.93–0.99; *χ* ^*2*^ = 10.09; *I* ^*2*^ = 80.2 %), 0.59 (95 % CI 0.48–0.69; *χ* ^*2*^ = 14.12; *I* ^*2*^ = 85.8 %) to 0.95 (95 % CI 0.88–0.98; *χ* ^*2*^ = 16.83; *I* ^*2*^ = 88.1 %) for EBUS-TBNA group and cTBNA group, respectively. Moreover, threshold effect analysis showed that their spearman correlation coefficients were respectively 0.949 (*P* = 0.051) and 0.400 (*P* = 0.600) for EBUS-TBNA group and cTBNA group, which suggested that no diagnostic threshold effect existed for histological diagnoses, and we believe that the heterogeneity among studies could mainly result from clinical and methodological differences.

**Fig. 5:**
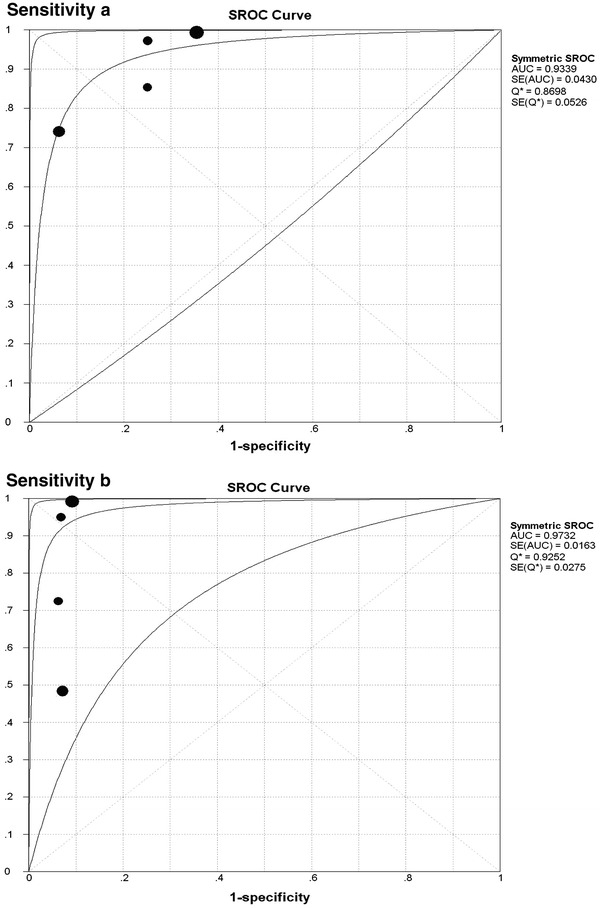
Summary receiving operating characteristic curve and Q* index for EBUS-TBNA (**a**) and cTBNA (**b**)

The pooled PLR, NLR, and DOR were 3.19 (95 % CI 2.03–5.00), 0.09 (95 % CI 0.01–0.67), and 75.38 (95 % CI 16.38–346.97) for EBUS-TBNA group, respectively. Correspondingly, the pooled PLR, NLR, and DOR were 11.11 (95 % CI 5.16–23.96), 0.11 (95 % CI 0.01–1.47), and 108.17 (95 % CI 13.84–845.35) for cTBNA group, respectively. Additionally, the two SROC were presented in Fig. 4, which showed that AUC and Q* index with a standard error (SE) were 0.9339 (0.8698 ± 0.0526) and 0.9732 (0.9252 ± 0.0275) for EBUS-TBNA group (Fig. 5a) and cTBNA group (Fig. 5b), respectively.

Specifically, the Z-test results suggested that the pooled sensitivity of EBUS-TBNA was significantly higher than that of cTBNA (*P* < 0.01), and the pooled specificity, DOR and Q* index of cTBNA were obviously higher than those of EBUS-TBNA (all *P* < 0.01).

### Subgroup analyses

Also, we performed subgroup analyses using a random effects model to explore the heterogeneity of sensitivity and examine the influence of various exclusion criteria based on sample sizes (>60 vs. ≤60), region (Asia vs. North America), and patients with diagnosis either sarcoidosis or other thoracic lesions. Table 2 showed the detailed indication for subgroup analyses of EBUS-TBNA and cTBNA for the pooled sensitivity, specificity and DOR in all eligible studies.

**Table 2:** Subgroup analyses of the eligible studies for the pooled sensitivity, specificity, and DOR based on various exclusion criteria

Various exclusion criteria	n/N	Pooled sensitivity (95 % CI), *I* ^*2*^		Pooled specificity (95 % CI), *I* ^*2*^		Pooled DOR (95 % CI), *I* ^*2*^	
		EBUS-TBNA	cTBNA	EBUS-TBNA	cTBNA	EBUS-TBNA	cTBNA
All included trials	440/4	0.90 (0.85–0.94), 90.2 %	0.76 (0.68–0.82), 95.1 %	0.75 (0.60–0.87), 47.6 %	0.94 (0.86–0.98), 0.0 %	75.38 (16.38–346.97), 0.0 %	108.17 (13.84–845.35), 44.9 %
Number of patients ≤60	110/2	0.93 (0.80–0.98), 71.4 %	0.82 (0.63–0.94), 77.1 %	0.79 (0.49–0.95), 0.0 %	0.96 (0.82–1.00), 0.0 %	47.12 (4.85–457.56), 0.0 %	93.64 (10.09–869.02), 0.0 %
Number of patients >60	330/2	0.89 (0.83–0.94), 96.3 %	0.74 (0.66–0.82), 98.2 %	0.73 (0.54–0.88), 80.3 %	0.93 (0.81–0.99), 0.0 %	110.89 (14.13–870.58), 0.0 %	122.09 (1.29–11574.71), 78.9 %
Patients from Asia	190/2	0.81 (0.70–0.89), 88.3 %	0.55 (0.43–0.67), 91.6 %	0.85 (0.62–0.97), 65.1 %	0.96 (0.81–1.00), 0 %	65.79 (7.94–545.02), 0.0 %	51.18 (2.43–1077.38), 48.1 %
Patients from North America	250/2	0.97 (0.91–0.99), 89.1 %	0.94 (0.86–0.98), 93.7 %	0.67 (0.45–0.84), 0.0 %	0.93 (0.81–0.99), 7.4 %	80.91 (5.04–1299.21), 35.9 %	224.23 (7.59–6627.62), 60.5 %
Patients with diagnosis or suspected of sarcoidosis	180/2	0.78 (0.67–0.87), 36.7 %	0.54 (0.43–0.65), 74.3 %	1.00 (0.63–1.00), 0.0 %	1.00 (0.75–1.00), 0.0 %	29.35 (3.20–269.08), 0.0 %	21.50 (2.63–175.73), 0.0 %
Patients with mediastinal lymph nodes	260/2	1.00 (0.96–1.00), 0.0 %	1.00 (0.95–1.00), 0.0 %	0.69 (0.52–0.84), 0.0 %	0.93 (0.83–0.98), 0.0 %	176.81 (21.51–1453.23), 0.0 %	614.46 (67.13–5624.23), 0.0 %

### Safety

From Table 1, we found that there were no obvious complications observed in two groups excluding rare patients with minor or moderate bleeding. Both EBUS-TBNA and cTBNA are safe and provide a well tolerated approach in the diagnosis of patients with mediastinal lymphadenopathy.

## Discussion

The current meta-analysis including 4 RCTs was conducted to critically evaluate the diagnostic value of EBUS-TBNA compared with cTBNA in patients with mediastinal lymphadenopathy. The results of our study indicated that both EBUS-TBNA and cTBNA are safe and provide good diagnostic value for patients with mediastinal lymphadenopathy. EBUS-TBNA with a higher sensitivity performs better than cTBNA, however, the pooled specificity, DOR and Q* index of cTBNA were obviously higher than those of EBUS-TBNA.

EBUS-TBNA has been a new approach in the estimation of thoracic disease not only in nonmalignant diseases such as sarcoidosis and tuberculosis but also in mediastinal lymphadenopathy, particularly in cases of malignancy (Garwood et al. 2007; Tournoy et al. 2010; Schmid-Bindert et al. 2013; Lee et al. 2014). Up to now, the latest guideline and expert panel report about the technical aspects of EBUS-TBNA was published on CHEST (Wahidi et al. 2015). The authors recommended that EBUS-TBNA be used for diagnosis in patients with suspected sarcoidosis, tuberculosis and even lymphoma. However, as well as the previous systematic reviews (Adams et al. 2009; Gu et al. 2009; Varela-Lema et al. 2009; Yang et al. 2014; Trisolini et al. 2015), they had a common point that they did not compare EBUS-TBNA with cTBNA. Additionally, even if they enrolled some trials combining EBUS-TBNA and cTBNA arms, these trials were non-RCT, such as retrospective or observational studies. Importantly, conclusions in terms of diagnostic yield cannot be drawn until further head-to-head evidence is available. Therefore, different from the aforementioned systematic reviews, we enrolled available RCTs combining EBUS-TBNA and cTBNA to critically assess the diagnostic value of EBUS-TBNA versus cTBNA in patients with mediastinal lymphadenopathy.

In the present meta-analysis, we mainly focused on evaluating the diagnostic value across EBUS-TBNA and cTBNA arms during the diagnosis of mediastinal lymphadenopathy, both in benign and malignant etiologies. Our results showed that the pooled sensitivity was 0.90 and 0.76, specificity was 0.75 and 0.94, DOR was 75.38 and 108.17, and AUC was 0.9339 and 0.9732 for EBUS-TBNA group and cTBNA group, respectively. Moreover, the Z-test results suggested that the pooled sensitivity of EBUS-TBNA was considerably higher than that of cTBNA, and the pooled specificity, DOR and Q* index of cTBNA were obviously higher than those of EBUS-TBNA. Based on the above results, we believed that our results could not be used as the basis to choose the operating mode of TBNA for the clinicians, and the choice needs to be followed the principle of individuation. Next, in the present study, sensitivity analyses did not obviously alter the heterogeneity among studies for the pooled sensitivity. The results from subgroup analyses indicated that EBUS-TBNA might be superior to cTBNA in patients from Asia and with diagnosis or suspected of sarcoidosis in terms of the pooled sensitivity. But further studies are needed to investigate these topics. Finally, threshold effect analysis showed that no diagnostic threshold effect existed for histological diagnoses, which indicated that the heterogeneity among studies could be seen as a result of clinical and methodological differences.

Additional areas of study are important for future clinical research. Advanced technology and equipment means more expense; therefore, cost-effective analyses are needed. Moreover, further segregation of the diagnostic yield in malignant versus benign disease is needed as it is difficult to delineate which approach is more effective for various patients (Yarmus et al. 2011; Jiang et al. 2014). This is especially true of lung cancer staging as there is no comparison of these two modalities with regard to accurately staging the mediastinum in patients with non small cell lung cancer. Therefore, future research should pay more attention to the tailoring principle of individuation. Additionally, performance experience and learning curve are considerable factors. While EBUS-TBNA may seem to be a more secure, attractive and dependable modality to the inexperienced bronchoscopist (Yarmus et al. 2011), the learning curves for both modalities were reported (Bellinger et al. 2014; Haponik et al. 1995; Hermens et al. 2008; Kemp et al. 2010; Mehta 2013; Wahidi et al. 2014). Next, the type of sedation was not the same settings in the four RCTs. Does less sedation for cTBNA make it more cost effective? Future research should focus on the important issue. Finally, cTBNA combining ROSE has been proven to have many benefits, such as improving the diagnostic yield, decreasing the number of needle passes, and reducing the need for additional diagnostic procedures (Diacon et al. 2005; Mondoni et al. 2013; Gasparini and Bonifazi 2014). However, ROSE was not used in the four RCT analyzed in our study, and it is unknown if the addition of ROSE would affect yield in a comparative study of these modalities. Further trials are warranted to compare the role of EBUS-TBNA with ROSE versus that of cTBNA with ROSE.

To be sure, several limitations exist in our study. First, the patients were heterogenous with a mixture of benign and malignant etiologies, and different size a location of the intrathoracic or mediastinal lymph nodes. Second, procedural aspects were also heterogenous, such as the number of aspiration passes, the type of needle, and the type of sedation. Operator experience level is variable which may affect the applicability of our results. Third, four RCTs with a wide variation in sample size were incorporated into our analysis. Overestimation of the diagnostic value is the most likely to occur in smaller than in larger studies. Finally, several unpublished or missing data may increase the risk of bias.

## Conclusions

In summary, this study suggests that both EBUS-TBNA and cTBNA are safe and provide diagnostic value for mediastinal and hilar adenopathy. Both have advantages and disadvantages. The optimal choice of procedure should be individualized based on availability, patient characteristics and operator experience. Further robustly designed RCTs are needed to better explore the current findings and continue to investigate the most appropriate diagnostic modality.
